# Identifying Recent Cholera Infections Using a Multiplex Bead Serological Assay

**DOI:** 10.1128/mbio.01900-22

**Published:** 2022-10-26

**Authors:** Forrest K. Jones, Taufiqur R. Bhuiyan, Rachel E. Muise, Ashraful I. Khan, Damien M. Slater, Kian Robert Hutt Vater, Fahima Chowdhury, Meagan Kelly, Peng Xu, Pavol Kováč, Rajib Biswas, Mohammad Kamruzzaman, Edward T. Ryan, Stephen B. Calderwood, Regina C. LaRocque, Justin Lessler, Richelle C. Charles, Daniel T. Leung, Firdausi Qadri, Jason B. Harris, Andrew S. Azman

**Affiliations:** a Department of Epidemiology, Johns Hopkins Bloomberg School of Public Health, Baltimore, Maryland, USA; b Infectious Diseases Division, International Centre for Diarrhoeal Disease Research, Bangladesh (icddr,b), Dhaka, Bangladesh; c Division of Infectious Diseases, Massachusetts General Hospital, Boston, Massachusetts, USA; d Laboratory of Bioorganic Chemistry, National Institute of Diabetes and Digestive and Kidney Diseases, National Institutes of Health, Bethesda, Maryland, USA; e Department of Medicine, Harvard Medical School, Boston, Massachusetts, USA; f Department of Immunology and Infectious Diseases, Harvard T.H. Chan School of Public Health, Boston, Massachusetts, USA; g Department of Epidemiology, University of North Carolina Gillings School of Global Public Health, Chapel Hill, North Carolina, USA; h University of North Carolina Population Center, University of North Carolina Gillings School of Global Public Health, Chapel Hill, North Carolina, USA; i Division of Infectious Diseases, University of Utah School of Medicine, Salt Lake City, Utah, USA; j Division of Microbiology and Immunology, University of Utah School of Medicine, Salt Lake City, Utah, USA; k Department of Pediatrics, Harvard Medical School, Boston, Massachusetts, USA; l Institute of Global Health, University of Geneva, Geneva, Switzerland; Harvard School of Public Health

**Keywords:** serosurveillance, multiplex bead assay, seroincidence, *Vibrio cholerae*

## Abstract

Estimates of incidence based on medically attended cholera can be severely biased. Vibrio cholerae O1 leaves a lasting antibody signal and recent advances showed that these can be used to estimate infection incidence rates from cross-sectional serologic data. Current laboratory methods are resource intensive and challenging to standardize across laboratories. A multiplex bead assay (MBA) could efficiently expand the breadth of measured antibody responses and improve seroincidence accuracy. We tested 305 serum samples from confirmed cholera cases (4 to 1083 d postinfection) and uninfected contacts in Bangladesh using an MBA (IgG/IgA/IgM for 7 Vibrio cholerae O1-specific antigens) as well as traditional vibriocidal and enzyme-linked immunosorbent assays (2 antigens, IgG, and IgA). While postinfection vibriocidal responses were larger than other markers, several MBA-measured antibodies demonstrated robust responses with similar half-lives. Random forest models combining all MBA antibody measures allowed for accurate identification of recent cholera infections (e.g., past 200 days) including a cross-validated area under the curve (cvAUC_200_) of 92%, with simpler 3 IgG antibody models having similar accuracy. Across infection windows between 45 and 300 days, the accuracy of models trained on MBA measurements was non-inferior to models based on traditional assays. Our results illustrated a scalable cholera serosurveillance tool that can be incorporated into multipathogen serosurveillance platforms.

## INTRODUCTION

Cholera remains a global public health threat with an estimated 95,000 deaths per year, especially in areas without safe water and adequate sanitation (1). Seventh pandemic strains of Vibrio cholerae (toxigenic serogroup O1, El Tor biotype) are responsible for most cholera cases, with endemic transmission in Africa and Asia as well as large outbreaks in conflict zones, humanitarian crises, and postdisaster settings (2–4). Several countries plan to achieve large reductions in cholera cases and deaths over the next decade using a multisectoral approach that includes the administration of oral cholera vaccines and investment in water and sanitation infrastructure (5). A clear understanding of the magnitude of pandemic V. cholerae transmission at the subnational level is essential for targeting and monitoring global progress toward ending cholera.

Cholera surveillance typically consists of clinic-based syndromic surveillance for acute watery diarrhea with infrequent laboratory confirmation (6). When laboratory confirmation is performed, often less than half of suspected cholera cases have detectable V. cholerae by culture, with considerable variation across settings (7–9). Because most V. cholerae infections lead to mild/no symptoms, clinical surveillance detects only a small fraction of infections (10, 11). Clinical surveillance systems are also subject to biases related to individual health care seeking patterns and the design of the surveillance system (e.g., sentinel sites) (12, 13). As a result, clinical surveillance alone provides a skewed understanding of disease burden and transmission of pandemic V. cholerae.

Serosurveillance has been a useful complement to clinical surveillance for a variety of pathogens and there is growing interest in its use for monitoring cholera incidence (14, 15). Despite variability in clinical outcomes, infection with cholera, regardless of symptoms, typically leads to a robust measurable immune response. This includes a rise and eventual decay in serum-circulating antibodies against multiple epitopes (10, 16). As a result, cross-sectional measurements of circulating antibodies can provide insights into the incidence and timing of past infections. The two most common methodologies used to measure antibodies generated in response to V. cholerae infection are the vibriocidal assay and enzyme-linked immunosorbent assay (ELISA). Previous studies have shown that vibriocidal titers rise quickly after infection and then decay toward preinfection levels after 1 year (16, 17). However, the vibriocidal method is a functional assay that requires culturing V. cholerae over several hours (thus requiring a Biosafety Level 2 facility) and is challenging to standardize across laboratories (18). Although ELISAs targeting immunoglobulin G (IgG) and IgA antibodies binding to known antigens are easier to implement, these assays are less predictive of recent infection than the vibriocidal assay (19). Previous work illustrated that combining vibriocidal titers with enzyme-linked immunosorbent assay (ELISA) antibody measurements in statistical models can identify recently infected individuals for estimating cholera seroincidence (i.e., the incidence of meaningful immunologic exposures to V. cholerae O1 over a specific period) (19).

Over the past decade, advances in high-throughput serological multiplex bead assays (MBAs) have enabled their use to study the burden, risk, and dynamics of a variety of pathogens (20–23). These assays only require a small volume of serum (e.g., 1 μL to measure multiple antigens compared to 12.5 μL for the vibriocidal assay [when performed in duplicate for both assays]), potentially are more sensitive (24), and could be easier to standardize (25). Additionally, they allow for the characterization of large numbers of antigens simultaneously, improving the efficiency and cost of the assay compared to running multiple ELISAs (26). This also facilitates broad exploration of novel antigens that may correlate with previous exposure or immunity. If measuring multiple antibodies to V. cholerae antigens is as predictive of recent infection as the vibriocidal assay, serosurveillance would be feasible in many more settings. However, the use of cholera antigens in an MBA to predict recent infection has not been previously assessed.

Here, we characterized postinfection antibody dynamics to seven cholera antigens up to 3 years postinfection in a cohort of confirmed medically attended V. cholerae O1 infections. These antigens included O1 serogroup Ogawa serotype O-specific polysaccharide (OSP, part of the LPS), O1 serogroup Inaba serotype OSP, cholera toxin B subunit (CT-B), cholera toxin holotoxin (CT-H), toxin coregulated pilus subunit A (TcpA), V. cholerae cytolysin (VCC) (also known as hemolysin A), and V. cholerae sialidase. We use these serological data to train statistical models to identify recently infected individuals. We then compare the performance of models based on this assay to those based on traditional antibody measurements and suggest a reduced panel of antigens to be used in MBA arrays for future cholera serosurveillance efforts.

## RESULTS

### Description of individuals and timing of samples.

We tested 296 samples from 48 confirmed cholera cases (4 to 1,083 days postinfection) and 9 samples from 3 uninfected household contacts of cases (Table S1 at the Extended Supplement). V. cholerae serogroup O1 was isolated from each case with most being serotype Ogawa (81%) and the rest being serotype Inaba. The median age of cases at the time of enrollment was 11 years (interquartile range [IQR], 6 to 26 years) with 17% being <5 years old and 35% being ≥18 years old. Most cases were male (62%) and nearly half had the O blood type (46%).

All cases had a baseline sample collected <5 days after infection. Nearly all cases had additional samples collected between 6 and 11 days (*n* = 46), 28 to 36 days (*n* = 46), 87 to 110 days (*n* = 42), and 172 to 191 days (*n* = 39) post infection. Between 268 and 1,083 days after infection, 1 person had three samples, 35 people had two samples, 2 people had one sample, and 10 people had zero samples collected (Fig. S2 at the Extended Supplement).

### Kinetics of biomarkers in confirmed cholera cases.

**FIG 1:**
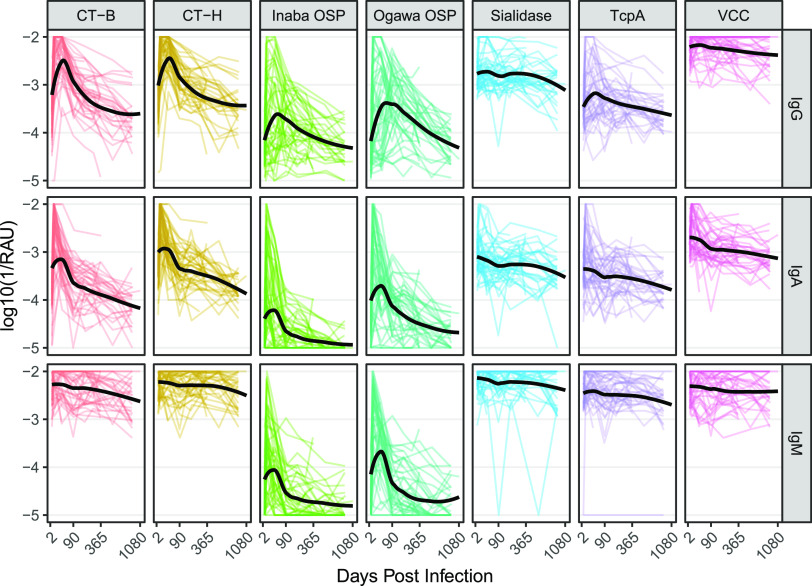
Antibody concentrations of IgG, IgA, and IgM against V. cholerae *O1* antigens among culture confirmed cholera patients. The *x*-axis is square root transformed. Each colored line indicates individual trajectories over time. The black solid line is a loess smooth function. Trajectory plots of other measured antigens can be found in Fig. S1. Similar plots with net MFI can be found in the Extended Supplement.

After infection, the levels of several V. cholerae O1-specific antibodies in most cases had a steep rise followed by variable decays (Fig. 1). Robust anti-CT-B, anti-CT-H, anti-Inaba OSP, and anti-Ogawa OSP antibody responses were observed across the study cohort (except anti-CT-B and anti-CT-H IgM). Individuals who had an increase in anti-sialidase, anti-TcpA, and anti-VCC antibodies tended to be adults. Among anti-CT-B, anti-CT-H, anti-Inaba OSP, and anti-Ogawa OSP antibodies, the observed median day of peak measurement were 8 days for IgA, between 8 and 63 days for IgM, and 25 to 33 days for IgG. As expected, there were no substantial increases in anti-O139 OSP or anti-influenza antibodies (used as a control) after infection (Fig. S1 in the Extended Supplement). Some antibody responses were highly correlated due to antigen homology, including cross-specificity between the similar Ogawa and Inaba OSP antigens, and the CT and LT antigens (27) (Fig. S3 in the Extended Supplement).

**FIG 2:**
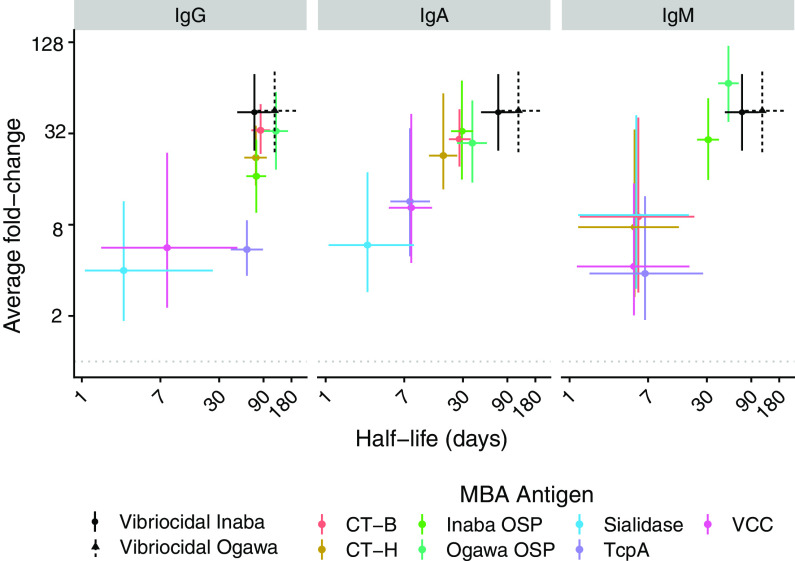
Estimated duration of half-life and average fold-change from exponential decay models. Each point indicates the median estimate of the average individual fold-rise from baseline to peak (*y*-value) and the median estimate of the half-life (*x* value) for exponential decay univariate models. Marginal 95% credible intervals are shown as lines. Model estimates for the vibriocidal assay are shown for reference and are identical across panels. A similar plot with Net MFI can be found in the Extended Supplement.

We fit a series of statistical models to estimate the population-level rise and decay of each marker. As biphasic models did not consistently fit better than exponential models (i.e., only in 12 of 39 markers did biphasic models fit significantly better [Extended supplement]), we assumed that antibody decay was exponential. These models were able to reproduce individual-level antibody trajectories (at Extended Supplement). The magnitude of antibody increase and duration of half-life varied considerably across MBA measures of antibody levels and vibriocidal titers (Fig. 2). The fold increase in vibriocidal titers (average 45-fold increase for Ogawa and 44 for Inaba) was higher than for all MBA-measured antibodies except anti-Ogawa OSP IgM. We estimated relatively large increases in anti-Ogawa OSP and anti-Inaba OSP antibodies across isotypes (range, average 17- to 69-fold rise) and large increases in anti-CT-B and anti-CT-H IgG and IgA antibodies (range, average 22- to 34-fold rise). Anti-Ogawa OSP IgG antibodies had the longest estimated half-life (122 days [95% CI, 91 to 165]), similar to that of vibriocidal Ogawa antibodies (118 days [95% CI, 74 to 201]). Anti-CT-B IgG, anti-CT-H IgG, anti-Inaba OSP IgG, anti-TcpA IgG, and vibriocidal Inaba antibodies had slightly shorter half-lives (60 to 84 days) while the half-life for IgA and IgM antibodies was generally shorter (range, 3 to 51 days). Antibodies measured by ELISA had less pronounced responses (average boosts were all <10-fold) and were relatively short-lived (half-lives were all <51 days).

Antibody kinetics differed by both age and infecting serotype (Fig S4 and Fig S5 at the Extended Supplement). We found that individuals <10 years old tended to have smaller anti-Ogawa and Inaba OSP IgG boosts (i.e., fold-increases were 3.6 (95% CI, 1.2 to 10.6) and 3.4 (95% CI, 1.2 to 9.3) times smaller) followed by slower decay (i.e., the difference in half-life was 175 (95% CI, 7 to 320) and 64 days (95% CI, -11 to 176) longer) compared to those ≥10-years old (see Extended Supplement). Individuals <10 years old had lower baseline and boosts for anti-Ogawa and anti-Inaba OSP IgA, but the rates of decay were similar. Individuals <10 years old had little difference compared to those ≥10 years old in their anti-OSP IgM and anti-CT-B trajectories for any isotype. Individuals ≥10 years old had an increase in anti-TcpA IgG that was 2.2 times higher (95% CI, 1.0 to 4.7) than those <10-years old. Individuals with Ogawa infections on average had 7.2 (95% CI, 2.0 to 23.7) times higher increases in anti-Ogawa OSP IgM, 3.6 (95% CI, 1.0 to 12.8) times higher increases in anti-Ogawa OSP IgG, and 2.8 (95% CI, 0.7 to 10.3) times higher increases in anti-Ogawa OSP IgA than those with Inaba infections (see Extended Supplement). On average, increases in anti-Inaba OSP IgG, IgA, or IgM were similar regardless of infection serotype. There were little differences in terms of baseline values, boost, or decay rates by O-blood group or sex (see Extended Supplement).

### Identification of recent infections with cross-sectional serologic measurements.

**FIG 3:**
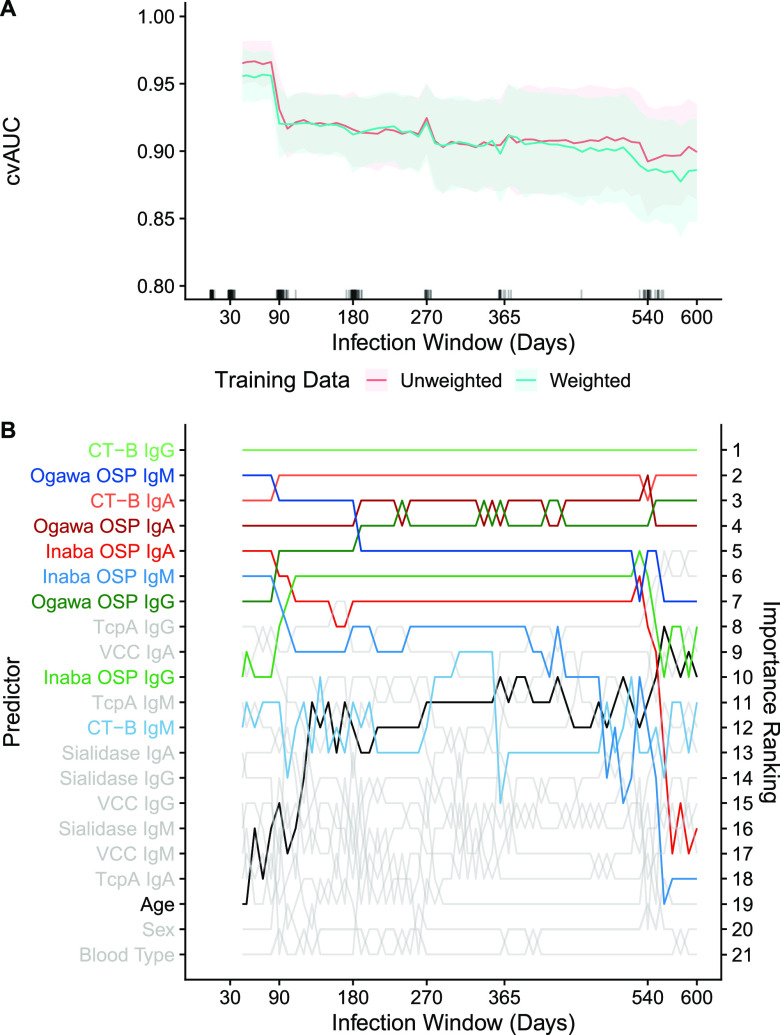
Cross-validated area under the receiver operating characteristic curve (cvAUC) and predictor importance rankings for MBA markers across random forest models with various infection windows. Estimates of mean cvAUC (10-fold) and 95% confidence interval are shown for weighted and unweighted models between 50- and 600-day infection windows at 10-day intervals (A). The rug plot shows the day of collection of samples from cases used in training models. Samples collected under 5 days since infection, over 600 days since infection, or from household contacts are not shown. For each infection window of weighted models, the rankings of predictors by their importance are shown on the *y*-axis (B). The colors of lines are unique to each predictor. A similar plot with net MFI can be found in the Extended Supplement.

We estimated receiver operator curves and their accompanying cross-validated area under the curves (cvAUCs) from random forest models trained on 18 MBA markers (three isotypes and six antigens) and three individual nonimmunological factors (age, sex, O-blood group) aimed at identifying recently infected individuals. Measurements of anti-CT-H antibodies were not included in these models given their high correlation with that of anti-CT-B antibodies. The average cvAUC was consistently above 89% regardless of infection window but was higher at shorter time windows (Fig. 3A and Fig. S6 at the Extended Supplement). Models using observations that were weighted based on time from infection either performed equally or slightly worse than models without weights. As an ensemble of four different machine learning models performed only slightly better than the random forest, with a great increase in complexity, we conducted further analyses with the random forest model alone (see Extended Supplement).

Across infection windows, anti-CT-B IgG antibodies were consistently the most influential predictor of recent infection while the relative importance of predictors changed with different infection windows (Fig. 3B and Fig. S6 at the Extended Supplement). With infection windows shorter than 90 days, anti-Ogawa OSP IgM was the second most influential marker, but waned in influence over longer windows, while the relative influence of anti-Ogawa OSP IgG increased over time. Anti-CT-B IgA and anti-Ogawa OSP IgA were consistently among the most influential markers. Anti-Ogawa OSP markers were always more influential than anti-Inaba OSP markers within the isotype (likely because 81% of cases used to train the models were infected with the V. cholerae Ogawa serotype). Other antibodies, age, sex, and blood type did not greatly influence the model.

**FIG 4:**
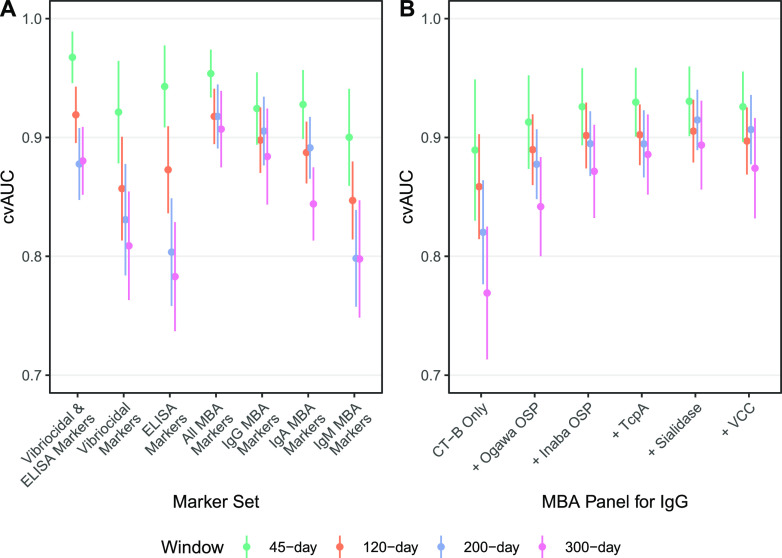
Comparison of cross-validated AUC across random forest models trained on traditional and MBA serological markers for 45-day, 120-day, 200-day, and 300-day infection windows. Random forest models were fit using a specified marker set and individual-level factors, including age, sex, and blood type (A). Estimated mean and 95% confidence intervals for cvAUC are reported. Models fit reduced panels of IgG MBA markers are shown where the antigen that is indicated (by a plus sign) is included in the model with all other antigens shown to the left of it. (B). The order of how antigens were added was determined by the variable importance when fitting a model with only IgG MBA markers. A similar plot with net MFI can be found in the Extended Supplement.

We then compared cvAUC from models that fit MBA measurements with those fit with the traditional vibriocidal and ELISA measurements for four infection windows (45-day, 120-day, 200-day, and 300-day). The model fit with both vibriocidal and ELISA markers was highly predictive of recent infection when considering 45-day (cvAUC, 97%), 120-day (cvAUC, 92%), 200-day (cvAUC, 88%), and 300-day (cvAUC, 88%) infection windows (Fig. 4A and see the Extended Supplement). The cvAUC of models trained with all 18 MBA markers was consistently similar to models trained with vibriocidal and ELISA markers (range of the ratio of the mean cvAUC_{MBA} to the mean cvAUC_{Traditional}, 0.99 to 1.05) (Fig. 4A).

### Simplifying the multiplex bead assay panel.

We explored how using fewer MBA markers would impact model performance (Fig. 4). Across all timescales, both a model using six IgG MBA markers (e.g., 200-day cvAUC, 91%; 1% lower mean cvAUC; Fig. 4B) and a model using six IgA MBA markers (e.g., 200-day cvAUC, 89%; 3% lower mean cvAUC) was higher than using six IgM MBA markers (e.g., 200-day cvAUC, 80%; 12% lower mean cvAUC).

Because many commonly used serosurveillance panels are based on IgG markers alone (28), we considered a reduced panel with only IgG (Fig. 4B). A model using only anti-CT-B IgG and nonimmunological predictors was predictive (200-day cvAUC, 82% [95% CI, 78% to 86%]) of recent cholera infection across all infection windows. Adding both Ogawa OSP and Inaba OSP led to additional improvement (200-day cvAUC, 89% [95% CI, 87% to 92%]). The addition of TcpA, VCC, and sialidase had little impact on the overall cvAUC (200-day model, 91% [95% CI, 88% to 94%]). Models without age, sex, and blood type had similar performance to their counterparts with these nonimmunological predictors.

**FIG 5:**
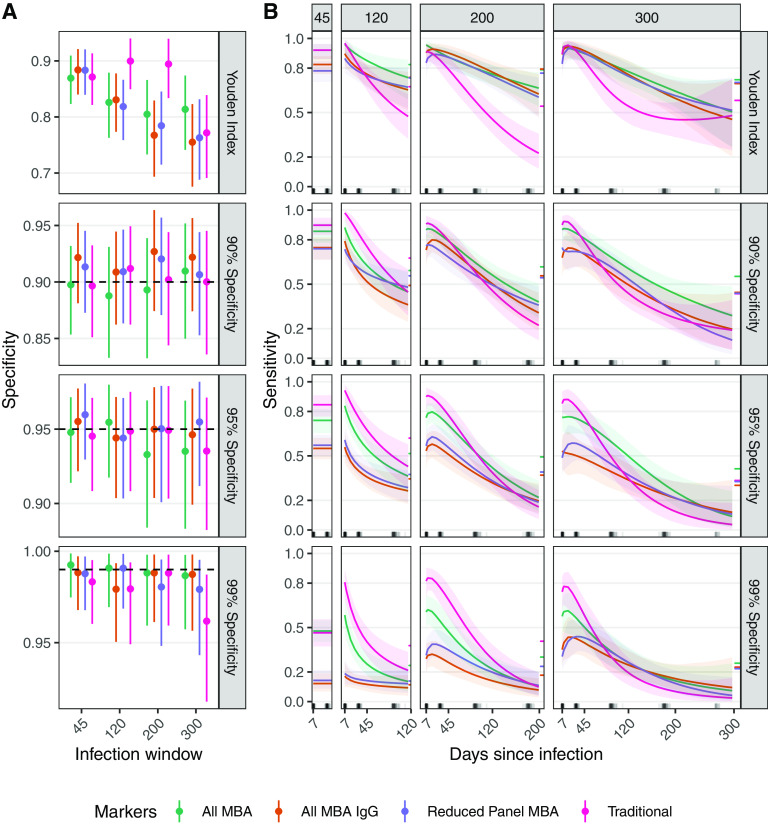
Specificity and time-varying sensitivity estimates of random forest models trained with leave-one-out cross-validation for 45-day, 120-day, 200-day, and 300-day infection windows using different cutoffs. Median and 95% credible intervals are shown for the estimated (A) nominal specificity (black dashed line) and (B) time-varying sensitivity. Each row represents a different method for acquiring a cutoff, including the Youden index or maximizing sensitivity for a desired value of specificity. The relationship between logit(sensitivity) and time since infection (log-transformed) was constant for the 45-day window, linear for the 120-day, quadratic for the 200-day window, and cubic for the 300-day window. Traditional = vibriocidal Ogawa, vibriocidal Inaba, and 4 ELISA markers, All MBA = 18 MBA markers, All MBA IgG = 6 MBA markers, Reduced panel = Ogawa OSP, Inaba OSP, and CT-B IgG. All models also included age, sex, and blood type as predictors. A similar plot with Net MFI can be found in the Extended Supplement.

We then estimated the specificity and time-varying sensitivity of random forest models fit with traditional and MBA markers using leave-one-out cross-validation (Fig. 5). When using the Youden Index (i.e., jointly maximizing sensitivity and specificity), median estimates of specificity were all below 90% (Fig. 5A). Sensitivity estimates were negatively correlated with specificity estimates and did not vary substantially between models with different marker sets when using the same infection window (Fig. 5B). When fixing specificity at 90%, the (non-time-varying) median sensitivity estimates ranged from 71% to 90% across different models for the 45-day infection window. As for other infection windows, sensitivity steadily decreased over time for the 120-day (range of median estimates, 35% to 98%), 200-day (range of median estimates, 21% to 93%), and 300-day (range of median estimates, 8% to 92%) window.

## DISCUSSION

We developed an MBA to measure antibody responses to V. cholerae infection and then characterized the performance of these to identify recent infections thus allowing us to estimate seroincidence. Among cholera patients, anti-OSP and anti-CT-B antibody increases as measured by the MBA were of higher magnitude and duration than responses measured by ELISA and were comparable in magnitude and duration to traditional vibriocidal titers. Models using MBA-measurements accurately identified individuals infected within 300 days before blood collection and were equally accurate as models trained on vibriocidal assay and ELISA data. Owing to its accuracy and scalability, the use of a V. cholerae-antigen MBA provides an opportunity to increase global cholera seroincidence data and can be incorporated into multipathogen serosurveillance systems without using a combination of traditional serologic assays.

We also measured a suite of potentially informative antibody responses that have not been assessed for their utility in estimating seroincidence. These markers included VCC, sialidase, and TcpA. Compared to OSP- and CT responses, antibody responses to VCC, sialidase, and TcpA were highly variable and in aggregate were of lower magnitude and short duration. These features limit the utility of these novel biomarkers for estimating disease incidence. However, this may have been due to the large number of children in our sample, who compared to adults, had a less robust antibody response to these antigens (Fig. S6). This is consistent with previous studies showing that repeated exposures to V. cholerae are required to consistently produce antibodies to TcpA (29) and that in an area of endemicity responses to V. cholerae sialidase are associated with increasing age (30).

Given the lack of consistent and durable responses to VCC, sialidase, and TcpA, it is not surprising that a simplified MBA assay, measuring only responses to CT-B and OSP performed similarly to a full suite of antigens. Taken together, these results suggest that with the increasing use of multipathogen integrated serosurveillance using MBAs, the inclusion of three additional antigen targets (CT-B, Ogawa OSP, and Inaba OSP) to larger panels could efficiently provide data on V. cholerae O1 infection rates. While many serosurveillance panels only measure IgG antibodies (28), advancements to simultaneously measure multiple isotypes could further improve the detection of recent infections, especially when seroincidence over a short infection window is of interest (31).

Our study has some limitations. First, we analyzed data from a cohort of patients with severe cholera in an area of high endemicity. Many infections with V. cholerae *O1* lead to mild disease, and these infections may lead to different postinfection antibody responses (32, 33), potentially leading to misclassification. Despite the small number of individuals included in this study, our samples were selected to be ‘representative’ of a larger previously published cohort (Fig. S7 at the Extended Supplement), thus enhancing the generalizability of our findings. Individuals in Bangladesh are also likely infected several times throughout their lives (34) with each successive infection acting to boost antibody levels to higher levels than would be observed in an otherwise immunologically naive population. Reassuringly, however, we previously demonstrated that models fit traditional markers on Bangladeshi patients performed well when used in North American challenge study volunteers (19). Finally, the extent to which vaccinated individuals may be classified as recently infected using this model is not known. As cholera vaccines are used more frequently in cholera endemic settings, further studies are needed to optimize seroincidence models in partially vaccinated populations. For MBA-based platforms in which additional antigens and isotypes can be readily added, the addition of biomarkers that distinguish vaccination from infection could be a critical component of estimating infection incidence and, when combined with other data, disease burden.

As large investments in cholera prevention and control measures are being made, serosurveillance is likely to be an important tool for tracking trends in incidence to better target interventions and measure their effectiveness in reducing infections. We showed that measuring responses to as few as three antibodies with MBA can identify individuals infected up to 1 year before, with similar precision as traditional serologic methods which rely on less scalable functional measures of immunity. While cholera-specific panels may be warranted in some locations, the inclusion of V. cholerae-specific beads in larger multipathogen MBAs being used across the world could lead to a better understanding of the epidemiology of cholera To achieve this, investments in infrastructure for population-based serosurveillance, including the application of multipathogen MBAs, can be made in areas where cholera is endemic. Such efforts will improve our access to key data to aid the fight against cholera.

## MATERIALS AND METHODS

### Study population.

As described previously, patients hospitalized at the icddr,b Dhaka hospital with culture-confirmed V. cholerae O1 infection were enrolled between 2006 and 2018 (35, 36). Cases were followed with blood sample collection for up to 3 years post enrollment. We approximated the infection day by taking the difference between the enrollment date and sample collection date, then adding the number of hours of symptomatic diarrhea before enrollment (range, 3 to 60 h), then adding 1.4 days for the incubation period (37). Household contacts of confirmed cases with no evidence in stool or serum of recent infection were also enrolled with blood and stool samples collected at 2, 7, and 30 days postenrollment of the initial cases. We limited our selection of household contacts to those that had no evidence of cultured V. cholerae from stool samples during follow-up. Data on nonimmunological variables of age, sex, and blood type were available for all participants. We selected a set of 305 samples from 51 individuals to test and compare to previously measured antibody responses (Fig. S2, Fig. S7, at the Extended Supplement).

### Serological testing and data processing.

Based on a review of the published literature on immune responses to V. cholerae infection (27, 38, 39), we selected seven known cholera-related antigens to investigate with a multiplex bead assay. These included O1 serogroup Ogawa serotype O-specific polysaccharide (OSP, part of the LPS), O1 serogroup Inaba serotype OSP, cholera toxin B subunit (CT-B), cholera toxin holotoxin (CT-H), toxin coregulated pilus subunit A (TcpA), V. cholerae cytolysin (VCC) (also known as hemolysin A), and V. cholerae sialidase. Additionally, O139 serogroup OSP (V. cholerae O139 serogroup has been rarely detected as circulating in the last decade, but is included in the most commonly used oral cholera vaccines), heat-labile enterotoxin subunit B (LT-B), and heat-labile holo-enterotoxin (LT-H) (expressed during infection with enterotoxigenic Escherichia coli [ETEC] and have a high degree of homology with cholera toxin counterparts) and influenza hemaglutinin 1 (flu) (as a control antigen) were also selected. Antigens were produced as previously described (29, 30, 40–43), or purchased from a commercial source, and are described in detail in the testing protocol. OSP antigens were produced as OSP:BSA (bovine serum albumin) conjugates to facilitate binding to the polystyrene beads (40–42). All antigens were conjugated to Luminex magnetic beads using carbodiimide coupling according to the manufacturer’s recommendations.

Each plate included a dilution series (from pooled convalescent-phase sera of 5 patients with culture-confirmed V. cholerae O1 infection) and control wells, all of which were run in triplicate. Following the testing protocol, serum, beads, and secondary antibodies binding to IgG, IgA, and IgM (Southern Biotech, catalog numbers 9040, 2050, and 9020) were added to each well. Samples were run on a Luminex Flexmap 3D machine at Massachusetts General Hospital by one technician. Bead counts and median fluorescence intensity (MFI) values were exported from the Exponent software program. Plates were retested when over half of the positive control dilutions had ≥5 antigens with a coefficient of variation (calculated from triplicate MFI measurements) greater than 20%.

For the analysis, any measurements with a bead count of less than 30 were excluded (<0.1%). MFI values were averaged across replicate wells. We standardized MFI values from the assay to help adjust for interplate variability by calculating the relative antibody unity (RAU) (Supplementary Methods) (44). For each plate, we fit a 4-parameter log-logistic model to the dilution series and used the median of parameter estimates to predict the RAU for each sample. (Supplementary Methods and Extended Supplement) (45, 46). For samples with a predicted RAU outside the range of 10^5^ and 10^2^, the RAU was set at the threshold value. Additionally, we calculated the Net MFI for each sample (i.e., MFI of the sample, MFI of blank well, but censored at 10 FI units). Despite some between-plate variability and limits of detection, we observed a high correlation between the Net MFI and RAU measurements, across time points. Thus, we conducted all primary analyses using RAU with corresponding Net MFI-based results shown in the Extended Supplement.

### Statistical analysis.

We fit hierarchical regression models for each marker to estimate the degree of antibody boosting postinfection and its decay rate after the boost for each serological marker. We used a Bayesian framework with two components, which included a kinetic model and a measurement model (Supplementary Methods; (47)). For the kinetic model, we assumed individuals had a linear rise in log concentration of antibodies from 5 days postinfection to an individual-specific peak followed by exponential decay over time. In the measurement model, we assumed random error was normally distributed (on the log scale) and accounted for the fact that some observations were censored (e.g., vibriocidal titers and RAU measurements at the interpolation boundaries). We fit the models using Markov Chain Monte Carlo methods implemented in Stan (46). We also estimated the association of age group (<10 years versus ≥10 years), sex (male versus female), blood type (O blood type versus non-O blood type), and infecting serotype (Ogawa versus Inaba), on baseline antibody levels, boosting, and decay. We also fit biphasic decay models and compared them with the exponential parameterization using the loo package (48) to calculate the difference in the expected log pointwise predictive density.

We explored the ability of statistical models to identify individuals who were recently infected with V. cholerae O1. We used four definitions of recent infection (i.e., infection windows) where infections occurred (i) 5 to 45 days, (ii) 5 to 120 days, (iii) 5 to 200 days, or (iv) 5 to 300 days before blood collection. Measurements from uninfected household contacts and cases infected <5 days prior (due to insufficient time to generate an antibody response) were always considered not recently infected. Using serological biomarkers and three nonimmunological demographic variables (age, sex, and blood type), we trained random forest classification models to identify recently infected individuals (49). We removed 11 (4%) of samples (from cases) for this analysis as they were either missing a vibriocidal or ELISA measurement. We fit models with weights to account for both class imbalance and the large concentration of measurements collected during the early convalescent period (7 to 30 days) compared to the later postinfection period (see Extended Supplement). Using 10-fold cross-validation, we estimated the cvAUC to evaluate the ability of the model to identify recently infected individuals. We kept all measurements from each individual within the same cross-validation fold. To understand which markers had the largest influence on model fits, we used a permutation importance metric (50). To understand how our model choice may have impacted our results, we fit three alternative models (i.e., lasso and elastic-net regularized generalized linear models, Bayesian additive regression trees, and extreme gradient boosting) and combined results from each to yield ensemble predictions and an accompanying cvAUC (51).

We also evaluated the specificity and time-varying (i.e., time since infection) sensitivity of the random forest classification models using leave-one-individual-out cross-validation. For each fold (i.e., left-out individual), we fit random forest models to 100 random samples of 50% individuals in the training set and found a cutoff that satisfies the Youden Index or the desired specificity cutoff (90%, 95%, or 99%) in the other 50% of individuals. Using the median value of these cutoffs and a model fit with the entire training data, we predicted the serostatus of the left-out samples. Using the predicted serostatus for each sample from leave-one-individual-out cross-validation, we fit hierarchical logistic regression models to estimate the specificity and time-varying sensitivity of each random forest model (52). For time-varying sensitivity models, we assumed that the logit(sensitivity) was a function of (log-transformed) days since infection (34). We allowed for increasingly complex functions as the time since infection increased, including a constant sensitivity for the 45-day model, a linear decrease in sensitivity for the 120-day model, a quadratic polynomial for the 200-day model, and a cubic polynomial for the 300-day infection window model.

### Ethics.

Original data collection was approved by the icddr,b Ethics Review Board and these analyses were deemed exempt from review by the Johns Hopkins Bloomberg School of Public Health Institutional Review Board.

### Data availability.

The extended supplement as well as data and code used to select samples and conduct primary analyses are available at https://github.com/HopkinsIDD/cholera-multiplex-panel. The lab protocol for the MBA assay is available at https://doi.org/10.17504/protocols.io.3byl4b1x8vo5/v1.
